# Heat-related cardiovascular mortality risk in Cyprus: a case-crossover study using a distributed lag non-linear model

**DOI:** 10.1186/s12940-015-0025-8

**Published:** 2015-05-01

**Authors:** Małgorzata J Lubczyńska, Costas A Christophi, Jos Lelieveld

**Affiliations:** Energy, Environment and Water Research Center, The Cyprus Institute, Nicosia, Cyprus; Cyprus International Institute for Environmental and Public Health in association with Harvard School of Public Health, Cyprus University of Technology, Limassol, Cyprus; Department of Environmental Health, Harvard School of Public Health, Boston, MA USA; Max Planck Institute for Chemistry, Mainz, Germany

**Keywords:** Temperature, Cardiovascular mortality, Climate change, Cyprus, Distributed lags, Case-crossover

## Abstract

**Background:**

The frequency and intensity of heat waves is projected to increase in many parts of the world, particularly in regions such as the Eastern Mediterranean and Middle East (EMME), where the warming trends are much larger than the global average. The relationship between air temperature and premature mortality is widely recognized, however, it is not well defined in the aforementioned region.

The objective of this study is to assess the relationship between cardiovascular mortality risk and air temperature in Cyprus, an island located centrally in the EMME.

**Methods:**

Daily cardiovascular mortality data and spatially aggregated daily mean, maximum, and minimum temperatures for the period 2004-2010 were analyzed using a case-crossover design combined with a distributed lag non-linear model.

**Results:**

A relationship between high temperatures and cardiovascular mortality was observed for cerebrovascular diseases, ischaemic and other heart diseases; this relationship was exacerbated on days with high temperatures. The highest relative risk was observed on the day of the heat event and remained significantly elevated for another day. The results were consistent regardless whether the minimum, maximum, or mean temperatures were used, although the association seems to be more pronounced with the mean temperatures, which suggests that consecutive high day- and night-time temperatures are the most hazardous.

**Conclusions:**

The identification of a positive relationship between high temperatures and cardiovascular mortality in Cyprus raises concerns. In view of the projected climate changes and strong increases in extreme heat events in the region, appropriate interventions need to be developed.

**Electronic supplementary material:**

The online version of this article (doi:10.1186/s12940-015-0025-8) contains supplementary material, which is available to authorized users.

## Background

The relationship between air temperature and premature mortality is widely recognized. Many studies, in various geographical locations, have reported that both spells of extremely low and extremely high temperatures can cause excess mortality [1-4]. However, differences in heat sensitivity, coping capacity, and adaptation measures of different populations, as well as climatic differences across the globe, can influence the relationship of air temperature with mortality and cause it to be region specific [3]. This implies that the relationship between air temperature and mortality, as reported for a certain region, cannot be directly extrapolated to other regions with, for example, different climatic zones, without introducing errors.

Following scientific consensus on the projected global warming, an overall substantial increase in air temperature, at least partially induced by humans, has been observed, while heat waves are very likely to increase in frequency and intensity in the future [5]. A recent study showed that the Eastern Mediterranean and Middle East (EMME) region is a climate change hotspot where temperatures increase more rapidly than elsewhere. For example, within the 20^th^ century, in many locations throughout the EMME region, warming trends of 0.4°C per decade were observed, a tendency that is much larger than the global average [6]. It is projected that this will continue in future and will be combined with a significant reduction of rainfall giving rise to a dramatic increase in hot weather extremes in most parts of the region [7].

These projections make it of paramount importance to develop a solid understanding of the relationship between temperature and mortality in the EMME region, despite alleviation in the increase in heat-related mortality observed over the course of the last century in various parts of the world [8-11]. This alleviation is mainly attributed to the implementation of adaptation measures, such as the wide use of residential air-conditioning systems. This is true in Cyprus as well where about 80% of households are equipped with one or more air-conditioning units [12]. However, despite of these measures, the mortality rate that is attributed to extreme heat events appears to be elevated.

In general, the incidence of heat-related mortality is the highest among the elderly and people with compromised health, according to studies originating mainly from the US, Europe, and Australia [13,14]. Cardiovascular diseases are among the main underlying causes of death during heat waves, defined as prolonged periods of unusually high air temperatures [2,13-17]. The present study focuses on heat-related cardiovascular mortality in the southern part of Cyprus, the area that is under the control of the Republic of Cyprus (hereafter referred to as Cyprus). The northern part of the island, where Republic of Cyprus has no effective control, is not included in this study.

The relation between high air temperatures and mortality in the EMME region has been a focus of interest in a limited number of studies. A significant association between temperature and total daily mortality has been established for the area of Greater Beirut [18]. This relationship has also been found to exist in Athens, Greece, where both cold and hot conditions were significant risk factors for daily natural cause mortality [19]. In an international study of temperature, heat and urban mortality clear evidence was found of increasing death rates from all non-external causes with increasing heat in the northern part of the EMME domain [20]. Another European study concluded that, in terms of health impact, the changing geographical patterns of high-impact heat waves will be most severe in the Mediterranean region which partly overlaps with the EMME region [16]. However, to our knowledge no study has focused specifically on heat-related cardiovascular mortality in the EMME region.

The objective of this research is to assess heat-related cardiovascular mortality in the EMME region using data from Cyprus, which geographically can be considered as the center of the region. Such direct approach is more appropriate than the use of existing models based on data from other parts of the world, such as the United States or Northern Europe, where heat sensitivity, coping capacity, adaptation measures, and climate conditions are different.

The present study is based on recent data, 2004-2010 time-series of air temperatures, and explicit categories of cardiovascular mortality in Cyprus.

## Methods

### Data collection

Cyprus is an island centrally located in the EMME region at about 35° north latitude and 33° east longitude. The geomorphology of Cyprus is marked by two mountainous areas; the Kyrenia range in the northern part of the island and the Troodos range largely in the southern part, with the highest elevation reaching 1952 m above sea level.

**Figure 1:**
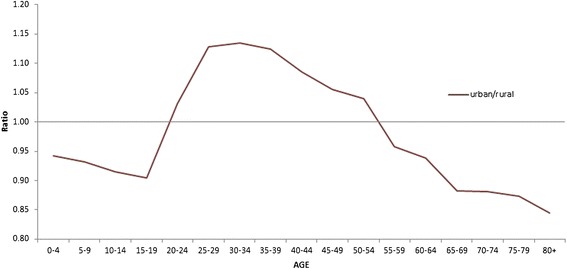
Urban-to-rural population ratio in Cyprus per age category, 2011 [21].

There are five main urban areas in the southern part of Cyprus, which account for approximately 67% of the total population of 840,400 inhabitants in 2011 [21]. The age-distribution of the population in the study area is not exactly proportional to the total population, with more children, adolescents and elderly living in the rural environment and people between 20 and 55 years old living in urban areas. However, these differences are not very profound (Figure 1).

#### Mortality data

The selection of the disease categories for inclusion in this study, with Cyprus as the study area, was based on the availability of data for the entire EMME region in the World Health Organization mortality database in order to maintain consistency with follow-up studies. The diseases for which records were available for each country within the EMME region were evaluated for their relevance with respect to temperature, based on expert opinion and a search of the literature. The final selection consisted of five categories of cardiovascular diseases, classified according to the 10^th^ revision of the International Classification of Diseases (ICD10): hypertensive diseases (I10-I13); ischaemic heart diseases (I20-I25); other heart diseases (I26-I51); cerebrovascular diseases (I60-I69); and remainder of diseases of the circulatory system (I71-I99) [22]. Daily mortality data on these five categories were then provided by the Ministry of Health in Cyprus for the period between 2004 and 2011 [23]. As the daily temperature time series available include data only until 31/12/2010, the study period in this research is limited to the period from 1/1/2004 until 31/12/2010.

Over this 7-year period, the total mortality related to the five categories of cardiovascular diseases considered in this study equaled 13,889 cases. The contribution per category to the total number of deaths is as follows: I10-I13 – 1220 cases (8.8%); I20-I25 – 4773 cases (34.4%); I26-I51 – 4595 cases (30.1%); I60-I69 – 2893 cases (20.8%); and I71-I99 – 408 cases (2.9%). No other relevant information aside from the date of death and the primary cause of death was provided in the database.

These diseases include anomalies in the blood flow. It is hypothesized that heat can further exacerbate these anomalies through vasodilation and/or dehydration, which could lead to hypovolemia [24,25]. Since blood transports oxygen to the organs, decreased blood flow could lead to insufficient oxygen supply throughout the human body which could cause organ damage, resulting, in the worst case, in premature death.

#### Meteorological data

The climate of Cyprus is predominantly subtropical, with daily mean summer temperatures ranging from 22°C in the high altitude areas to 29°C in the low altitude areas and daily maximum summer temperatures varying between 27°C and 36°C, respectively. The daily mean winter temperatures vary between 3°C and 10°C and the daily minimum winter temperatures between 0°C and 5°C in the high and low altitude areas, respectively [26].

The meteorological data used in this study originated from the Department of Meteorology of the Ministry of Agriculture in Cyprus. Daily minimum (T_min_), maximum (T_max_) and mean (T_mean_) temperatures were used in our analysis where T_min_ and T_max_ are daily (24-hour) observed extremes between 8:00am and 7:59am. Therefore T_min_, which generally occurs early in the morning, has a 1-day calendar day delay. Since T_mean_ combines the highest and the lowest measured temperatures within the 24-hour window, it can be seen as a representative value for consecutive day- and night-time temperature.

**Figure 2:**
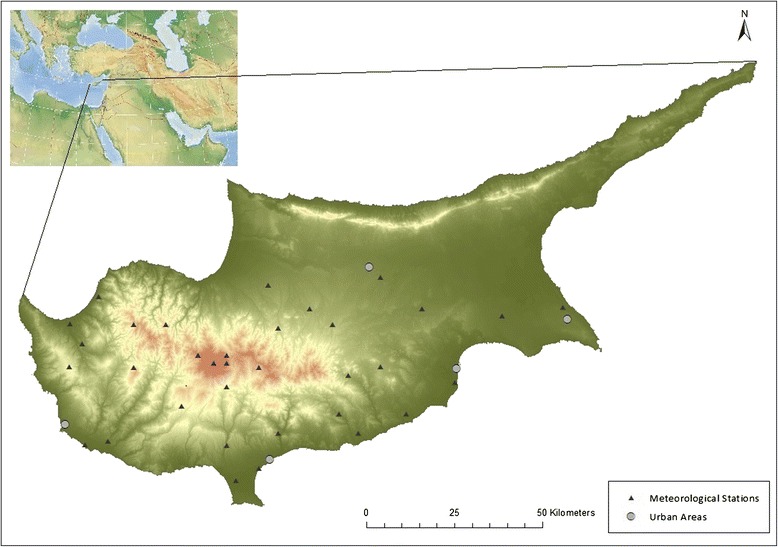
Distribution of meteorological stations and the main urban areas.

The measurements were taken at 34 weather stations distributed across Cyprus and located at various altitudes (Figure 2). As the available mortality time series database lacks spatial association and is characterized merely by one daily mortality value per disease for the entire study area, the daily temperature records were spatially averaged in order to create one representative daily value for the whole area. In order not to lose the spatial association of the temperature measurements, spatial averaging was based on weighing according to the population density, assuming that mortality is proportional to the population size: (Additional file 1). The population data were obtained from the Statistical Service of Cyprus [21].

The extent to which humidity plays a role in the temperature-mortality relationship is not entirely clear. Some studies suggest that humidity is an important component in the above mentioned relationship as it plays an essential role in the human thermoregulation, while other studies report a lack of such dependence [2]. Since the role of humidity in the temperature-mortality relationship is not well recognized and also because of the poor quality of the humidity database available, this study focused only on the association of temperature with cardiovascular mortality.

### Data analysis

The analysis of the relationship between temperature and cardiovascular mortality was carried out by applying a distributed lag non-linear model (DLNM) to a case-crossover design. A DLNM estimates the non-linear and the delayed effects of temperature on mortality using cross-basis functions to describe this relationship along the dimensions of temperature and lag simultaneously. These functions can be chosen independently for the two dimensions permitting a flexible fit [27,28].

The case-crossover design is a special case of a time-series analysis, wherein long-term and seasonal trends are controlled for by design through the use of relatively short time windows, namely, one calendar month in this study [27].

Applying a DLNM to a case-crossover design permits a flexible assessment of the non-linear and delayed effects of temperature on mortality and corrects for the effects of seasonality by design [27]. In order to combine the DLNM with the case-crossover design, a Poisson regression model was used, which allows for overdispersion:

$$ {Y}_t \sim Poisson\ \left({\mu}_t\right) $$

$$ \log\ \left({\mu}_t\right) = \alpha + \beta {T}_{t,l} + \lambda Strat{a}_t + \eta DO{W}_t $$

where *t* is the day of the observation; *Y*_*t*_ is the mortality count on day *t*; *α* is the intercept; *T*_*t*,*l*_ is the matrix that is obtained when the DLNM is applied to temperature; *β* is a vector of coefficients for that matrix; and *l* is the lag in days. *Strata*_*t*_ represents the case-control strata, a variable for the year and month that is used to control for long-term and seasonal trends; *λ* is a vector of coefficients and *DOW*_*t*_ is the day of the week on day *t*, with *η* being a vector of coefficients [27].

As the cross-basis functions to describe the temperature-response relationship can be independently chosen for temperature and lag, a quadratic basis spline was used for temperature and a natural cubic spline was used for the lag. In the quadratic basis spline, 4 degrees of freedom were used, and the knots were by default located at evenly distributed percentiles. For the natural cubic spline 4 degrees of freedom were selected and the boundary knots were by default placed at the range of the predictor, here lag 0 and lag 10. The choices of the degrees of freedom were based on the literature [27,29] and optimized with modified Akaike and Bayesian information criteria (QAIC and QBIC) [28]:

$$ QAIC = -2L\left(\widehat{\theta}\right)+2\widehat{\varnothing}k\ \mathrm{and}\  QBIC = -2L\left(\widehat{\theta}\right)+ \log (n)\widehat{\varnothing}k $$

where *L* is the log-likelihood of the fitted model; $\widehat{\theta}$ represent the parameters of the fitted model; $\widehat{\varnothing}$ is the estimated overdispersion parameter; *k* represents the total number of parameters; and *n* is the total number of observations.

The model that minimizes these two criteria was selected as the final model. For the purpose of this study, the “dlnm” package of R software was used [28,30]. The DLNM uses one value of temperature as the reference for the estimates that are expressed in terms of relative risk. The median temperatures for each of the three daily temperature series used in this study, i.e. T_max_, T_mean_ and T_min,_, were selected as these reference values and they equaled 25.9°C, 20.0°C, and 14.1°C, respectively (Table 1).

**Table 1:** **Values corresponding to reference temperature, the 90** ^**th**^ **, 95** ^**th**^ **and 99** ^**th**^ **percentiles and to the upper limit of each temperature distribution are presented**

	**T**_**mean**_ **[°C]**	**T**_**max**_ **[°C]**	**T**_**min**_ **[°C]**
Reference values	20.0	25.9	14.1
90^th^ percentile	28.4	34.6	22.2
95^th^ percentile	29.4	35.6	23.3
99^th^ percentile	31.2	37.4	25.3
Upper limit	33.6	39.9	27.9

Three-dimensional diagrams were produced that present the relationships between temperature and the different categories of cardiovascular mortality at the total range of lags, in terms of relative risk, as compared to the risk at the reference temperature. Plots showing the effect of a specific temperature and lag values on aggregated cardiovascular mortality are also presented. Since the focus of this study is on the right tail of the temperature distribution, the selected temperatures are all above the reference value and represent the 90^th^, 95^th^, and 99^th^ percentiles of the distribution. Furthermore, the maximum value of each temperature series is added to the selection (Table 1). In the analysis, the maximum temperature was rounded down to the largest integer value within each temperature range. Rounding down instead of up was used to prevent the integer from falling outside the recorded temperature range. The temperatures corresponding to the 90^th^, 95^th^, and 99^th^ percentiles of the time series fall within the recorded temperature range and were therefore rounded up.

In the sensitivity analysis, the maximum lag was changed to 5, 15, and 20 days. In addition, the window length of the calendar month in the case-crossover design was changed to 30, 28, and 21 days. The results were analyzed by comparing the estimated relative risks at 90^th^, 95^th^, and 99^th^ percentiles of the temperature distribution. The estimated relative risks are presented along with the corresponding 95% CI.

## Results

**Figure 3:**
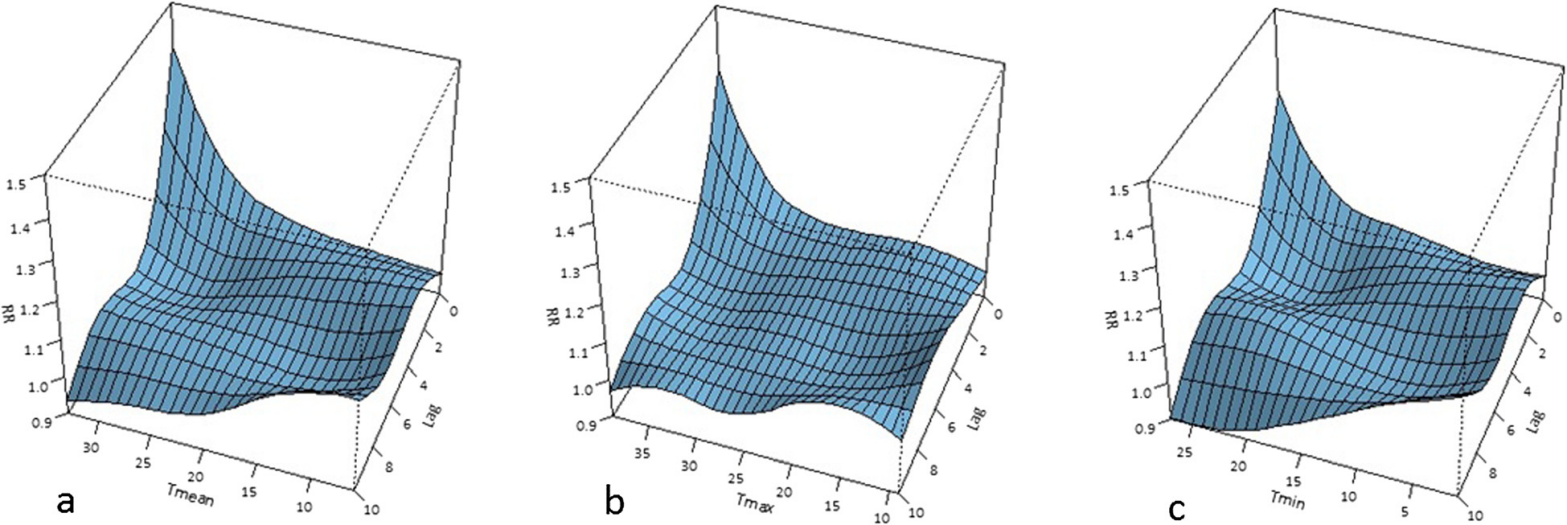
Relationship between temperature and relative mortality risks (RR) of aggregated cardiovascular diseases for lags ranging from 0 to 10 days for: **(a)** T_mean_; **(b)** T_max_; and **(c)** T_min_.

Figures 3a-c present the relationship between different temperature time series and aggregated cardiovascular mortality (i.e., aggregated over all five selected categories of diseases) for different lags. The graphs indicate a substantial increase in relative mortality risk (RR) for the highest temperatures between lag 0 and lag 2 with the largest increase occurring on the actual day of the event (lag 0). An interesting observation is that the relative mortality risk on the actual day of the event was larger when using the daily mean temperatures (Figure 3a) than when using the daily maximum temperatures (Figure 3b). The analysis with daily minimum temperatures (Figure 3c) showed the lowest increases in relative risk as compared to the other two temperature time series.

**Figure 4:**
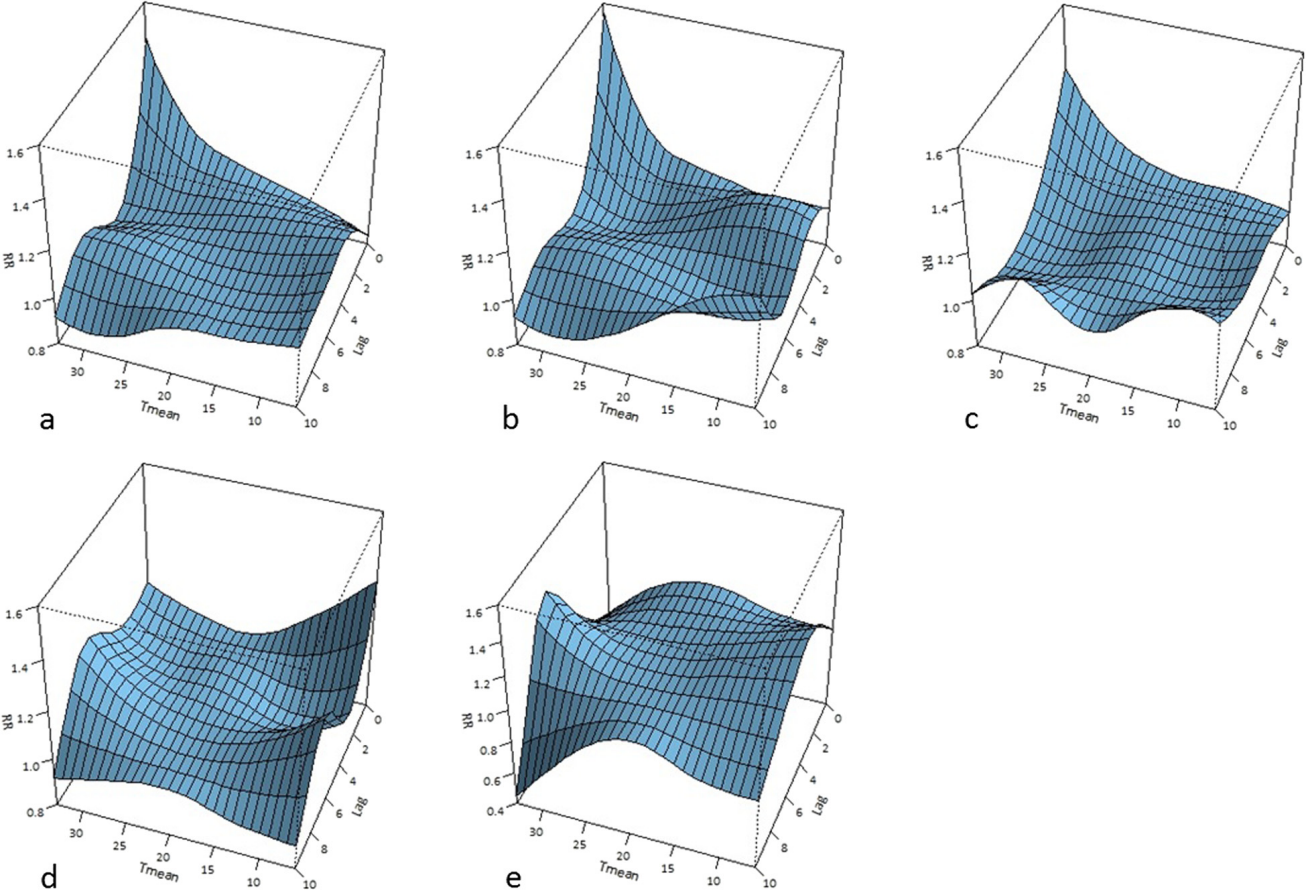
Relationships between temperature and relative mortality risk associated with the five categories of cardiovascular diseases. Legend: Relationship between T_mean_ and relative mortality risks of: **(a)** cerebrovascular diseases (ICD-10: I60-I69); **(b)** ischaemic heart diseases (ICD-10: I20-I25); **(c)** other heart diseases (ICD-10: I26-I51); **(d)** hypertensive diseases (ICD-10: I10-I13); and **(e)** remainder of diseases of circulatory system (ICD-10: I71-I99) for lags ranging between 0 and 10 days.

Figures 4a-e present the association of mean temperature and cardiovascular mortality attributed to the different diseases. Very similar patterns were identified in the analyses with T_max_ and T_min_. Only the results obtained with T_mean_ are shown here as these presented the largest increases in relative risk. The relationships of T_mean_ with cerebrovascular diseases (Figure 4a), ischaemic heart diseases (Figure 4b) and other heart diseases (Figure 4c) show similar patterns as the aggregate ones (Figure 3a) with the increase in relative risk on the actual day of the event being the largest for ischaemic heart diseases, followed by cerebrovascular diseases, and then other heart diseases. On the other hand, hypertensive diseases (Figure 4d) and the remainder of the diseases of the circulatory system (Figure 4e) show a rather random behavior.

**Figure 5:**
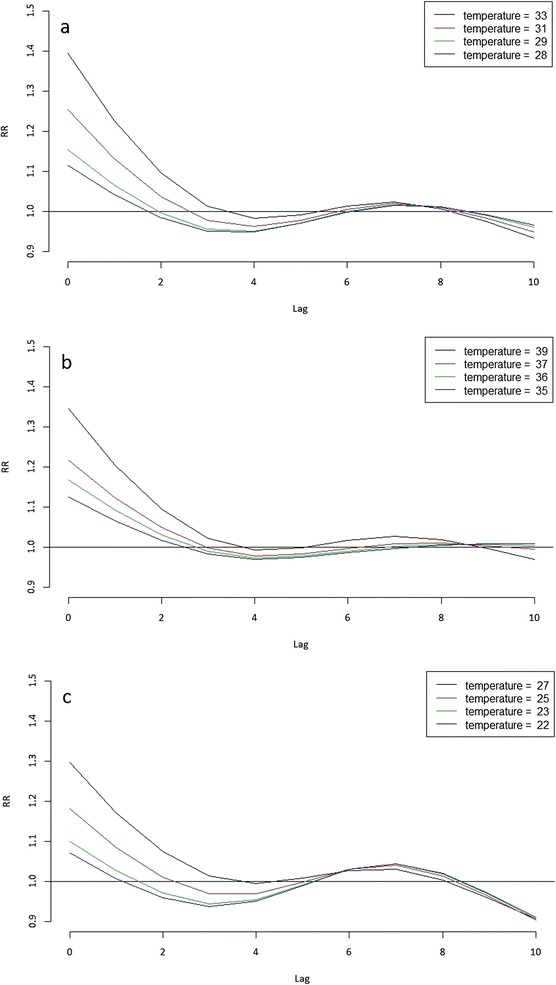
Lag-specific relative mortality risk associations for aggregated cardiovascular diseases. Legend: Lag-specific relative mortality risk (RR) associations for aggregated cardiovascular diseases at the temperatures corresponding to the 90^th^ (black), 95^th^ (red) and 99^th^ (green) percentiles of the temperature distribution and the maximum value (blue) of the temperature range for: **(a)** T_mean_; **(b)** T_max_; and **(c)** T_min_.

Figures 5a-c illustrate the lag-specific associations for aggregated cardiovascular diseases for the three temperature time series, T_mean_, T_max_, and T_min_, respectively. The pattern exhibited throughout the different ranges is very similar in all three cases, with T_mean_ showing slightly higher increases in relative mortality risk than T_max_ and T_min_. Furthermore, a drop in relative risk around lag 3 and 4 in all three temperature analyses is observed, indicating a possible mortality displacement, also known as a harvesting effect [31]. Around lag 5, the relative risk is elevated again and remains above 1 until approximately lag 9.

**Figure 6:**
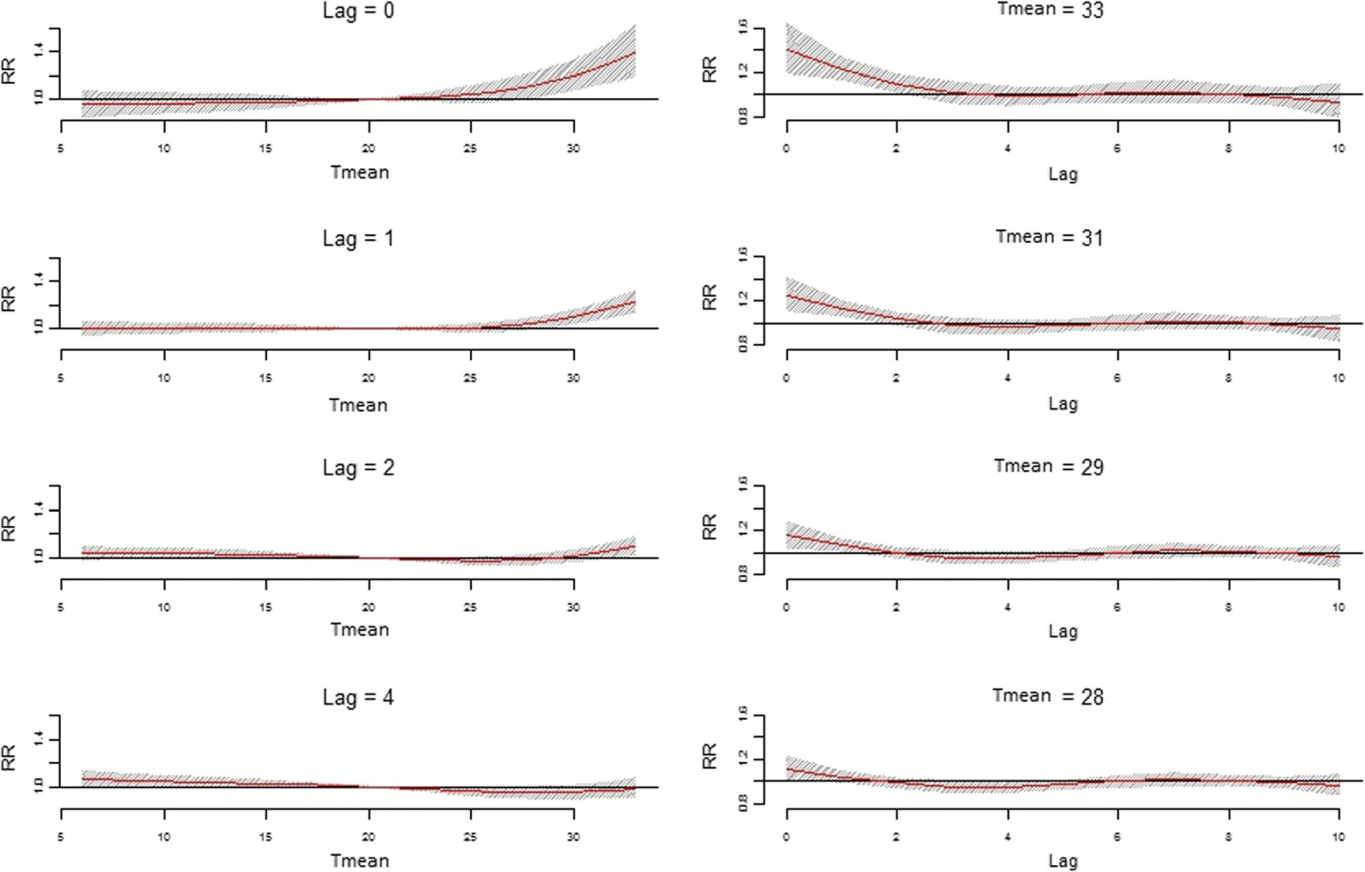
Relative cardiovascular mortality risk associations along the range of temperature and lags. Legend: The relative mortality risk (RR) associations for aggregated cardiovascular diseases along the range of T_mean_ at lags 0, 1, 2 and 4 (left column) and along the range of lags 0 to 10 at temperatures corresponding to the maximum value of the T_mean_ range and the 99th, 95th and the 90th percentiles (right column). The grey shadings represent the 95% confidence intervals.

In order to analyze the effect of a specific temperature and lag values on aggregated cardiovascular mortality more closely, the associations along the temperature ranges at lags 0, 1, 2, and 4 are plotted (Figure 6, left column); the associations along the range of lags (0 to 10) for the temperatures corresponding to the maximum value of the temperature range and the 99^th^, 95^th^, and 90^th^ percentiles are also presented (Figure 6, right column). The grey shadings represent the 95% CI. Only the results for T_mean_ are presented because of the higher relative risks associated with T_mean_ and the large similarity with the results obtained with both T_max_ and T_min_. The strong and significant increase in relative risk at lags 0 and 1 can be clearly seen in the figures, with the largest increase associated with the highest temperatures. Lags 2 and 4 show no significant effects. These results are consistent with T_max_ and T_min_ results (data not shown).

In addition to the case-crossover design, the DLNM was applied to a time-series design using a natural cubic spline with 7 df/year to model the seasonal and long-time trends, including a factor for the day of the week. The results of that analysis were very similar to the results shown before, with overall slightly higher relative risks (data not shown). We focused on the case-crossover design since it removes any confounding by fixed characteristics as every case serves as its own control. Moreover, the case-crossover design controls for long-term and seasonal trends through a relatively short time windows, here a calendar month [27].

In order to find the model with the best fit, the number of the degrees of freedom was changed from 4 to 6 for both; temperature and lag. The combination that minimizes the Akaike and Bayesian information criteria, here the combination of 4df for temperature and 4df for lag, was selected as the final set-up (Additional file 2: Table S1).

### Sensitivity analysis

Changing the maximum lag to 5, 15, and 20 days gave similar results when compared to the results obtained with the maximum lag set to 10 days (Additional file 3: Table S2). Furthermore, changing the window length of the calendar month in the case-crossover design to 30, 28, and 21 days lead to similar results as compared to the original set-up (Additional file 4: Table S3). Therefore, we are convinced that the model used in this study is capable of capturing the main effects of high temperatures on cardiovascular mortality sufficiently.

## Discussion

Despite the fact that no major differences were observed between the results using different temperature time series, the relative risk was highest for T_mean_ (Figures 3a and 5a). This suggests that the combination of high daytime temperatures with high temperatures at night is most harmful in the relationship between temperature and cardiovascular mortality. This is in line with findings of previous studies that argued that the thermoregulatory system of the human body can cope with extremely high temperatures as long as it has a sufficiently long period to cool down to normal temperatures following the hot period [15,32,33]. Consecutive high day and night time temperatures though prevent the human body from recovering from the heat stress.

In this study, the lowest increase in relative risk was associated with T_min_. However, the extensive presence of household air-conditioning in Cyprus [12] might mask the actual effects of high night time temperatures on mortality risk, especially considering that the majority of the population stays indoors during the night hours. No information on the actual usage of air-conditioning in Cyprus was available, and since the prevalence does not necessarily reflect the actual use, the above statement cannot be regarded as conclusive.

The relationship of T_mean_ with cerebrovascular diseases (Figure 4a), ischaemic heart diseases (Figure 4b), and other heart diseases (Figure 4c) show very similar patterns compared to the results wherein all diseases in aggregate are included (Figure 3a). Given the prevalence of these diseases, as reported earlier, this is expected, as the three categories combined equal almost 90% of the total deaths included in this study.

On the contrary, hypertensive diseases (Figure 4d) and the remainder of the diseases of the circulatory system (Figure 4e) show a rather random and not significant behavior. This is likely due to the limited amount of data for these two groups and/or due to the possibility that these two disease categories are less influenced by temperature compared to the other three categories of diseases.

The relative risk on the actual day of the event is highest for ischaemic heart diseases, followed by cerebrovascular diseases and other heart diseases. These results imply that among the categories of the diseases studied here, people with ischaemic disorders are the most vulnerable to extremely high temperatures.

The lag specific associations at different temperatures show similar results for all three temperature time series (Figure 5a-c) with the highest relative risks at lag 0, lasting up to lag 2. In addition, the three plots imply decreasing relative risks around lag 3 and 4, indicating a possible harvesting effect. However, upon closer inspection (Figure 4), this protective effect around lag 3 and 4 does not seem to be significant. The lack of significance of the results beyond lag 2 is evident in the analyses with all three temperature time series. This also holds true for the elevated relative risk between lag 5 and lag 9.

A limitation of the study is the lack of additional information about the cases, in particular their age, not allowing the use of a direct standardization method to account for the changing age-structure of the population, giving rise to potential bias due to the higher vulnerability of ageing populations to heat stress [2].

Moreover, due to the lack of information about the age of the cases, the spatial averaging of the temperature was based on weighing according to the population density without taking the age-structure into account. Since the age-structure is not exactly proportional to the overall population, this might lead to inaccuracy wherein urban meteorological records are given more weight, and therefore more influence in the final temperature time series due to denser population in the urban areas, while the vulnerability to heat-related cardiovascular mortality of that population is lower than the vulnerability of rural populations considering that people aged 20 to 55 years are over-represented in urban areas compared to rural areas. However, as it can be seen in Figure 1, these differences in age-structure between urban and rural areas in Cyprus are not very profound. Moreover, the temperature measurements are derived from 34 weather stations distributed fairly homogeneously throughout the study area (Figure 2) and therefore it can be assumed that taking into account the minor disproportionalities in the age-structure of the populations when weighing the temperature in order to obtain a spatial average would have only a minor impact on the final temperature time series.

## Conclusion

A relationship between high temperatures and cardiovascular mortality was observed for cerebrovascular diseases, ischaemic and other heart diseases, with the highest risk associated with ischaemic heart diseases. The relationship is strongest on the actual day of the event and the relative risk remains significantly elevated for approximately one day following the event. The increase in risk is most evident on days with the highest temperatures. The highest relative risks are observed for the daily mean temperature time series, which suggests that consecutive high day- and night-time temperatures are the most hazardous.

The identification of this relationship in Cyprus raises concerns especially in light of the climate projections indicating a large increase in hot weather extremes in the region. The findings of this study will be used in a follow-up study with the goal of establishing and testing a function representing the past temperature- mortality relationship and future projections for the entire EMME region, under the hypothesis that such a function established for Cyprus will be more representative of the EMME region than the functions used for other parts of the world.

With its focus on Cyprus, a part of a geographical region that is under-represented in the existing literature on heat-related mortality, and its focus on specific categories of cardiovascular diseases, this study adds new knowledge to the existing literature. Moreover, the identification of susceptible populations to heat-related cardiovascular mortality can improve the choice and implementation of adaptation measures and therefore facilitate the prevention and control of heat-related harmful effects of climate change.

## Additional files

Additional file 1:
**Weighting factors of the meteorological data** **[** 34 **].**
Additional file 2: Table S1. QAIC and QBIC for different combinations of temperature df and lag df. The model with the best fit is indicated in bold. Additional file 3: Table S2. Results from the sensitivity analysis. Relative risk (RR) values corresponding to three different temperature percentiles are shown, as well as the accompanying 95% CI. Additional file 4: Table S3. Results from the sensitivity analysis. Relative risk (RR) values corresponding to three different temperature percentiles are shown, as well as the accompanying 95% CI.

## Abbreviations

EMME: East Mediterranean and Middle East
°C: Degrees celsius
ICD10: 10^th^ revision of the International Classification of Diseases
T_max_: Maximum temperature
T_mean_: Mean temperature
T_min_: Minimum temperature
DLNM: Distributed lag non-linear model
CI: Confidence interval
