# Supplementary material for: Heat-related cardiovascular mortality risk in Cyprus: a case-crossover study using a distributed lag non-linear model
Source: Environ Health. 2015 May 1;14:39. doi: 10.1186/s12940-015-0025-8 (PMC4432944; doi:10.1186/s12940-015-0025-8)
Supplement: Additional file 1: — Weighting factors of the meteorological data [ 34 ]. [file 12940_2015_25_MOESM1_ESM.docx]

**Additional file 1**

*Weighting factors of the meteorological data*

For each village that is listed in the statistical service database, one or more meteorological stations were identified that are closest to the center point of that village. In cases where more than one station was identified, the difference in the elevation between the identified stations and the village center played a role. The final selection of a station to be associated with the given village was done according to the following two criteria: (i) the difference in the distances between each identified station and the village center is less than 5000 m and (ii) the difference in the elevations between each identified station and the village center is at least 200 m [34].

Weather stations that do not fulfill these selection criteria for any village included in the statistical service database were excluded from further analysis. The population of all villages attributed to a station following the above criteria was summed and divided by the total population of the study area. These are the weighting factors specific for each selected weather station that are applied to the corresponding temperature records, which are subsequently added to give one weighted numeral for temperature in the entire study area.
