# Supplementary material for: Heat-related cardiovascular mortality risk in Cyprus: a case-crossover study using a distributed lag non-linear model
Source: Environ Health. 2015 May 1;14:39. doi: 10.1186/s12940-015-0025-8 (PMC4432944; doi:10.1186/s12940-015-0025-8)
Supplement: Additional file 2: Table S1. — QAIC and QBIC for different combinations of temperature df and lag df. The model with the best fit is indicated in bold. [file 12940_2015_25_MOESM2_ESM.doc]

**Additional file 2: Table S1**: QAIC and QBIC for different combinations of temperature df and lag df. The model with the best fit is indicated in bold.

| Model | Temp df | Lag df | QAIC | QBIC |
| --- | --- | --- | --- | --- |
| 1 | 5 | 4 | 11545.27 | 12191.12 |
| 2 | 5 | 5 | 11551.92 | 12227.51 |
| 3 | 5 | 6 | 11559.17 | 12264.63 |
| 4 | 6 | 4 | 11548.74 | 12218.16 |
| 5 | 6 | 5 | 11557.84 | 12263.44 |
| 6 | 6 | 6 | 11565.45 | 12306.50 |
| **7** | **4** | **4** | **11541.95** | **12164.34** |
| 8 | 4 | 5 | 11549.92 | 12196.85 |
| 9 | 4 | 6 | 11554.20 | 12224.58 |
