# Supplementary material for: Heat-related cardiovascular mortality risk in Cyprus: a case-crossover study using a distributed lag non-linear model
Source: Environ Health. 2015 May 1;14:39. doi: 10.1186/s12940-015-0025-8 (PMC4432944; doi:10.1186/s12940-015-0025-8)
Supplement: Additional file 3: Table S2. — Results from the sensitivity analysis. Relative risk (RR) values corresponding to three different temperature percentiles are shown, as well as the accompanying 95% CI. [file 12940_2015_25_MOESM3_ESM.doc]

**Additional file 3: Table S2**: Results from the sensitivity analysis. Relative risk (RR) values corresponding to three different temperature percentiles are shown, as well as the accompanying 95% CI.

| temp | lag=5 |  | lag=10 |  | lag=15 |  | lag=20 |  |
| --- | --- | --- | --- | --- | --- | --- | --- | --- |
| percentile | RR | 95%CI | RR | 95%CI | RR | 95%CI | RR | 95%CI |
| 90^th^ | 1.036433 | 0.8840539 - 1.215078 | 1.021219 | 0.8495787 - 1.227537 | 0.9842638 | 0.8028105 - 1.20673 | 0.9767461 | 0.7851649 - 1.215073 |
| 95^th^ | 1.128557 | 0.9554864 - 1.332977 | 1.109122 | 0.9150044 - 1.344423 | 1.0566327 | 0.8526595 - 1.3094 | 1.0156986 | 0.805864 - 1.280171 |
| 99^th^ | 1.389631 | 1.139858 - 1.694135 | 1.357239 | 1.0720832 - 1.718241 | 1.2597745 | 0.960153 - 1.652895 | 1.1178229 | 0.8262658 - 1.512259 |
