# Supplementary material for: Heat-related cardiovascular mortality risk in Cyprus: a case-crossover study using a distributed lag non-linear model
Source: Environ Health. 2015 May 1;14:39. doi: 10.1186/s12940-015-0025-8 (PMC4432944; doi:10.1186/s12940-015-0025-8)
Supplement: Additional file 4: Table S3. — Results from the sensitivity analysis. Relative risk (RR) values corresponding to three different temperature percentiles are shown, as well as the accompanying 95% CI. [file 12940_2015_25_MOESM4_ESM.doc]

**Additional file 4: Table S**3: Results from the sensitivity analysis. Relative risk (RR) values corresponding to three different temperature percentiles are shown, as well as the accompanying 95% CI.

| temp | calendar month | | 30 days window | | 28 days window | | 21 days window | |
| --- | --- | --- | --- | --- | --- | --- | --- | --- |
| percentile | RR | 95%CI | RR | 95%CI | RR | 95%CI | RR | 95%CI-low |
| 90^th^ | 1.021219 | 0.8495787 - 1.227537 | 1.175073 | 0.9757459 - 1.415119 | 1.054991 | 0.869384 - 1.280223 | 1.118351 | 0.8999141 - 1.38981 |
| 95^th^ | 1.109122 | 0.9150044 - 1.344423 | 1.277699 | 1.0505698 - 1.553932 | 1.167884 | 0.9517107 - 1.43316 | 1.240404 | 0.985342 - 1.561491 |
| 99^th^ | 1.357239 | 1.0720832 - 1.718241 | 1.549244 | 1.2166827 1.972706 | 1.496144 | 1.1592744 - 1.930904 | 1.585222 | 1.1963238 - 2.100542 |
